# Effect of magnesium ions/Type I collagen promote the biological behavior of osteoblasts and its mechanism

**DOI:** 10.1093/rb/rbz033

**Published:** 2019-10-30

**Authors:** Xiaojing Nie, Xirao Sun, Chengyue Wang, Jingxin Yang

**Affiliations:** 1 Department of Prosthodontics, The Second Affiliated Hospital of Jinzhou Medical University, Jinzhou 121000, China; 2 College of Robotics, Beijing Union University, Beijing 100000, China

**Keywords:** magnesium ion, integrin, Type I collagen, FAK/ERK

## Abstract

Type I collagen (Col I) is a main component of extracellular matrix (ECM). Its safety, biocompatibility, hydrophilicity and pyrogen immunogenicity make it suitable for tissues engineering applications. Mg^2+^ also control a myriad of cellular processes, including the bone development by enhancing the attachment and differentiation of osteoblasts and accelerating mineralization to enhance bone healing. In our studies, Mg^2+^ bind collagen to promote the proliferation and differentiation of osteoblasts through the expression of integrins and downstream signaling pathways. In order to clarify the biological behavior effect of 10 mM Mg^2+^/Col I coating, we performed 3-(4,5-Dimethylthiazol-2-yl)-2,5-diphenyltetrazolium bromide (MTT), alkaline phosphatase (ALP), 4′6-diamidino-2-phenylindole (DAPI), Alizarin red staining and Rhodamine B-isothiocyanate (RITC)-labeled phalloidin experiments and found that 10 mM Mg^2+^ group, Col I-coating group, 10 mM Mg^2+^/Col I-coating group, respectively, promoted the proliferation and differentiation of osteoblasts, especially 10 mM Mg^2+^/Col I-coating group. We detected the mRNA expression of osteogenic-related genes (Runx2, ALP and OCN, OPN and BMP-2) and the protein expression of signaling pathway (integrin α2, integrin β1, FAK and ERK1/2), these results indicated that 10 mM Mg^2+^/Col I coating play an critical role in up-regulating the MC3T3-E1 cells activity. The potential mechanisms of this specific performance may be through activating via integrin α2β1-FAK-ERK1/2 protein-coupled receptor pathway.

## Introduction

Extracellular matrix (ECM) protein plays an important role in tissues repair and replacement [1]. Type I collagen (Col I) is a main component of ECM and performs structural and cell adhesion in many important organs and tissues. Col I is the main structural protein of all vertebrates. It is a natural polymer material and one of the main components of connective tissue. Its influence on medical applications has been confirmed, and it have been widely used in surgical sutures, anticoagulant materials, artificial blood vessels, skin, cartilage, etc. [2, 3]. And Col I was the most relapsing embedding substrate for cell encapsulation due to its biocompatibility, biodegradability and interconnected porous architecture and similarity to the natural ECM but collagen itself did not induce mineral formation and needed to exist and possessed poor load bearing capability [4–7].

However, magnesium is an essential element of the human body. Its modulus of elasticity is about 45 GPa, which is close to human bone and can effectively reduce stress shielding. As a degradable implant material, magnesium does not cause acute reactions after implantation. No obvious inflammatory reaction is found during implantation, which can meet the mechanical strength required for bone bearing area [8, 9], and after degradation, magnesium ions not only regulate cell behavior but also stimulate local bone formation and healing [10, 11]. Mg^2+^ is involved in bone development by enhancing the attachment and differentiation of osteoblasts and accelerating mineralization to enhance bone healing. However, Mg^2+^ concentration plays a key role in regulating bone formation, which exhibits concentration-dependent behavior and bone regeneration was associated [12]. Wang et al. showed that 6–10 mM Mg^2+^ promoted adhesion and proliferation of osteoblasts. Among them, 10 mM Mg^2+^ significantly promoted the adhesion and differentiation of osteoblasts, and 18 mM Mg^2+^ significantly inhibited the proliferation and differentiation of osteoblasts [13]. In particular, magnesium ions increase the affinity of integrins to ligands including ECM at a certain concentration [14]. Moreover, Mg^2+^ also control a myriad of cellular processes, including the functional properties of integrins which play a major role in anchoring cells to the ECM [15].

Integrins are widely expressed cell surface receptors that couple the interaction of the ECM with the cytoskeleton and transduce mechanochemical signals through the plasma membrane to initiate biological responses and play an important role [16]. Studies have shown that Col I interacts with the integrin β1 receptor and integrin α2 receptor on the cell membrane and mediates extracellular signals into cells. Col I induces osteoblasts differentiation, and integrin α2 and integrin β1-Col I interaction induces many cellular phenomena such as collagenase activation and induces collagen gel contraction. The integrin α2 and integrin β1-Col I interaction plays an important part in bone cell differentiation. Moreover, integrin α2β1 plays an important role in bone metastasis and integrin α2β1 mediates signaling pathway through activating p38 and ERK mitogen-activated protein kinases (MAPKs) and its signaling mediates cell proliferation, differentiation and cell death in various cell types including pre-osteoblasts [17]. Integrins are thought to mediate the extracellular environment by acting as a direct link between the two [18].

Therefore, this provided a new idea for the compounding of magnesium-based materials. Magnesium-based metal/Col I materials are able to combine the advantages of both. It is likely to be a very promising material to provide a basis for faster growth of osteoblasts. In order to prove the hypothesis, 100 μg/ml Col I (Solarbio, China) and 10 mM MgCl_2_ with glacial acetic acid add to medium, we determined that this experiment was performed by simulating magnesium-based metal/Col I materials *in vitro* environment and explored the effect Mg^2+^/Col I to promote the biological behavior of osteoblasts and its mechanism.

## Materials and methods

### Col I coating

**Figure 1:**
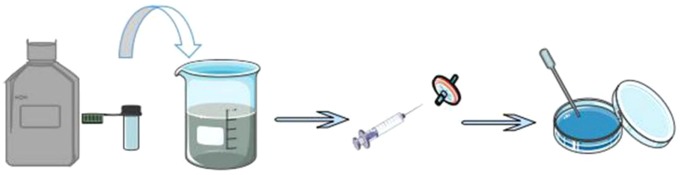
The process of Col I coating: Col I dissolved with glacial acetic acid-filtered coating plates for several hours at 37°C

Col I (Solarbio, China) was made to 100 μg/ml with glacial acetic acid (>99.5% Analytical purity, China) and the surface of the plates was coated at 100 μg/cm^2^. The plates were allowed to stand at room temperature or 37° C for several hours or at 2–8°C overnight. We aspirate excess liquid and allowed the dish to dry overnight (Fig. 1). The surface of the cell culture dish can be washed with a sterile balanced salt solution before inoculation of the cells.

### Preparation of magnesium ion and cell culture

Magnesium chloride (anhydrous MgCl_2_, 99.99%, Sigma-Aldrich, USA) was dissolved in deionized water and was filtered through a 0.22 μm filter (Corning, USA), and then diluted into cell culture medium formulated 10 mM Mg^2+^. The PH hasn't changed. The experiment was divided into A: intact a-MEM (Hyclone, USA) medium control group; B: 10 mM Mg^2+^ treatment group; C: Col I-coating treatment group and D: 10 mM Mg^2+^/Col I-coating treatment group. Osteoblast-like MC3T3-E1 cells (Institute of Basic Medical Sciences, Beijing, China) were cultured in a cell culture medium at 37°C, 5% CO_2_ and containing 10% fetal bovine serum (FBS, Invitrogen, Carlsbad, CA) and medium was changed once in every 2–3 days.

### Cell proliferation

The proliferation of MC3T3-E1 cells were determined by 3-(4,5-Dimethylthiazol-2-yl)-2,5-diphenyltetrazolium bromide (MTT). Cells were inoculated into a 96-well plate at a concentration of 1 × 10^3^/ml, and 2 ml of the cell suspension was added to each well, and five replicate wells of each group were cultured for 1, 3 and 5 days. Each empty medium was then aspirated and were rinsed with PBS and 10 µl cells of MTT (Solarbio, M8180, China) were added to each well. After 4 h of incubation in 5% CO_2_ incubator at 37°C, the medium was replaced with 150 μl of dimethyl sulfoxide to dissolve formazan. The plate was shaken for 10 minutes and then the solution in each well was transferred to a 96-well ELISA plate. The optical density (OD) of the dissolved solute was measured using an ELISA reader (Tecan, Austria) at 570 nm (*n* = 5 in each group).

### Cell apoptosis

The cells were inoculated into a 24-well plate at a concentration of 1 × 10^4^/ml, and 1 ml cells suspension was added to each well. Each group were cultured for 3 days. After 3 days, the cells were washed three times with PBS, fixed with 4% paraformaldehyde for 30 minutes and then washed three times with PBS, and added with 5 μg/ml of 4′6-diamidino-2-phenylindole (DAPI) reagent (Beyotime, China). After 3–5 minutes of staining, the PBS were washed three times. Images were taken under a microscope.

### Cell adhesion

Cells were seeded in confocal dishes at 1 × 10^4^/ml, and 1 ml of cell suspension was added to each group with three replicate wells per group. About 24 hours after cell attachment, the medium was aspirated and the cells were washed twice with 37°C and prewarmed 1X PBS (pH 7.4), fixed with 10% paraformaldehyde for 10 minutes at room temperature, washed 2–3 times with PBS for 10 minutes at room temperature. It was then permeabilized with a 0.5% Triton X-100 solution for 5 minutes. The cells were washed 2–3 times with PBS for 10 minutes at room temperature and 200 µl of the TRITC-labeled phalloidin (YEASEN, China) working solution was added and incubated at room temperature for 30 minutes in the dark, to wash three times with PBS for 5 minutes each time. The nucleus was counterstained with 200 µl of DAPI solution (Beyotime, China) for about 30 seconds and then was observed under a confocal microscope. Cell areas were measured using Image J software.

### Alkaline phosphatase activity

The viability of MC3T3-E1 cells were tested by alkine phosphatase (ALP, NanJing JianCheng Bioengineering Institute, A059-2, China). The cells were inoculated into a 24-well plate at a concentration of 1 × 10^4^/ml, and 1 ml of the cell suspension was added to each well, and five replicate wells of each group were cultured for 3, 5 and 7 days. The culture medium was carefully removed and the plates were gently washed twice with PBS. About 500 μl of 0.2% (v/v) Triton X-100 (Sigma, USA) was added to each well. The activity of the alkali phosphatase in the lysate was measured by adding an ALP kit according to the instructions and the absorbance OD at a wavelength of 520 nm using a spectrophotometer.

### ECM mineralization experiment

The cells were randomly added to the above experimental design at a concentration of 1 × 10^4^/ml. The cells were cultured in six-well plate and a cell suspension of 1 ml was added to each well with a pipette. Three replicate wells were set in each group. The cell culture plates were placed in a 37°C incubator with a CO_2_ concentration of 5%. After 18 days of culture, the supernatant was discarded and washed three times with PBS buffer then fixed with 95% alcohol for 30 minutes. Cells were washed again with PBS buffer for three times to remove residual wine and 3 ml of 0.1% alizarin red dye solution (Solarbio, G1452, China) was added to each well and incubated in a 37°C incubator for 30 minutes. After that, cells were washed with PBS buffer and were observed under a microscope.

### Western blot

MC3T3-E1 cells were cultured in α-MEM complete medium containing 10% FBS for 7 days, the cultured cells were lysed by using IP (Beyotime, China). The protein concentration was then measured by BCA protein quantification kit (Beyotime, China). The protein samples were heated and dried at 98°C for 5 min for denaturing the protein and loaded into SDS-PAGE. The proteins were transferred to the suitable of poly(vinylidene fluoride) (PVDF) membranes. The membranes were incubated in blocking solution (5% BSA) for 2 hours at room temperature, followed by washing with TBST lotion. After washing, the anti-FAK, anti-integrin a2, anti-integrin β1 and anti-ERK1/2 antibody were added and the membranes were incubated overnight at 4°C. Subsequently, the membranes were incubated with secondary antibody for 90 minutes at room temperature. After washing with TBST, the reaction was performed with a chemiluminescent reagent and exposure was performed. The western blot images were semi-quantitatively analysed by using Image J.

### Real-time quantitative PCR analysis

The expression levels of osteogenesis-related genes were evaluated on the basis of a real-time polymerase chain reaction (real-time quantitative PCR). The cells were seeded with 1 × 10^5^ cells/well. After culturing for 7 days. The total RNA was isolated using the Trizol reagent (Ambion, USA). Here, 1 mg RNA from each sample was reversed transcribed into complementary DNA using the Prime Script^TM^ RT reagent kit (Vazyme, USA). The forward and reverse primers for the selected genes were the same as those described in the literature. The expression levels of osteogenesis-related genes, including Alkine phosphatase (ALP), Runt-related transcription factor 2 (Runx2), Bone Morphogenetic Protein 2 (BMP-2), Osteocalcin (OCN) and Osteopontin (OPN), were quantified on the basis of real-time PCR (Bio-Rad iQTM5 multicolor real-time PCR detection system) with ChamQ^TM^Universal SYBR^®^qPCR Master Mix (Vazyme, USA). The internal reference gene was β-actin. Data analysis was carried out using theiQTM5 optical system software version 2.0 (Table 1).

**Table 1:** Sequence of primers used for RT-PCR analysis

Target	Forward primer	Reverse primer
BMP-2	AACACCGTGCGCAGCTTCCATC	CGGAAGATCTGGAGTTCTGCAG
ALP	CCAGAAAGACACCTTGACTGTGG	TCTTGTCCGTGTCGCTCACCAT
Runx-2	CCTGAACTCTGCACCAAGTCCT	TCATCTGGCTCAGATAGGAG
OCN	TGAGAGCCCTCACACTCCTC	ACCTTTGCTGGACTCTGCAC
OPN	GAGATTTGCTTTTGCCTGTTTG	TGAGCTGCCAGAATCAGTCACT
β-Actin	GGACTATGACTTAGTTGCGTTAC	TTTGCATTACATAATTTACACGA

### Statistical analysis

To analyse each group with at least three samples, all data were statistically analysed using one-way ANOVA. Comparisons were evaluated between statistically significant differences between sample groups. Quantitative data are presented as the mean ± standard deviation for each group. *P* values < 0.05 was considered statistically significant.

## Results

### Collagen I coating

#### Cell proliferation

**Figure 2:**
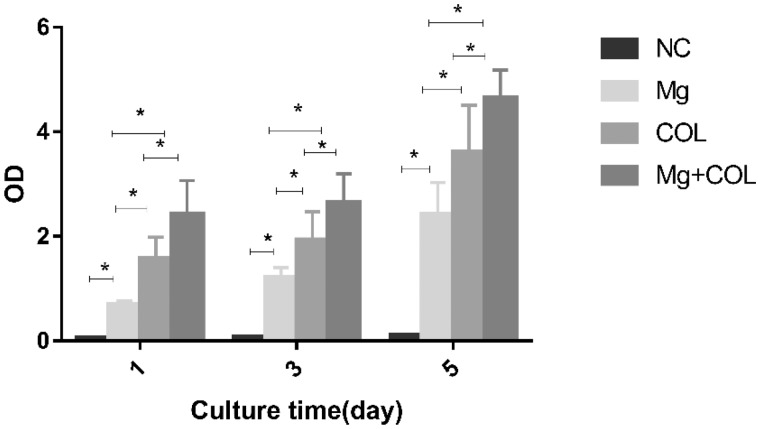
OD values of NC (control group), Mg (10 mM Mg^2+^ group), COL (Col I-coating group), Mg+COL (10 mM Mg^2+^/Col I-coating group) after incubation of MC3T3-E1 cells for 1, 3 and 5 days. One-way ANOVA (*n* = 5 per treatment group). *P < 0.05 versus control group

We cultured MC3T3-E1 under four different culture conditions including NC (control group), Mg (10 mM Mg^2+^ group), Col I (Col I-coating group), Mg+Col (10 mM Mg^2+^/Col I-coating group) and divided into three group in which cells were cultured for 1, 3 and 5 days. Cells were compared for cell proliferation by MTT assay. Compared the Col I-coating group, MC3T3-E1 cells proliferated faster in Col I-coating group than the 10 mM Mg^2+^ group. The results showed that the proliferation of the Col I-coating group was higher than that of the 10 MmMg^2 +^ group, suggesting that Col I promoted the proliferation of MC3T3-E1 cells at the same time and conditions. However, compared with the Col I-coating group, we also found that the proliferative capacity of MC3T3-E1 cells was significantly improved in the 10 MmMg^2^^+^/Col I-coating group (Fig. 2).

### Cell apoptosis

**Figure 3:**
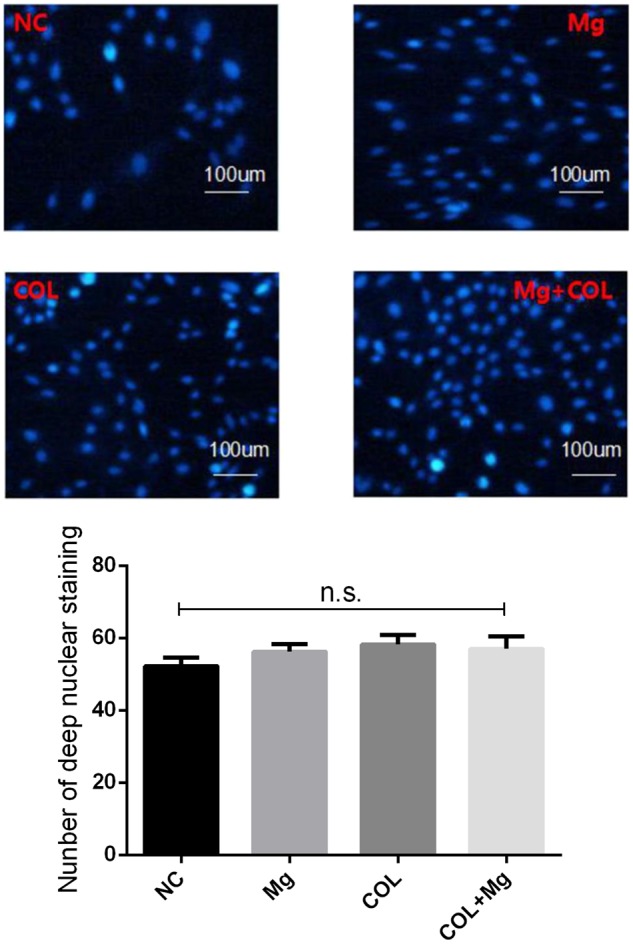
The DAPI staining results for the NC (control group), Mg (10 mM Mg^2+^ group), COL (Col I-coating group) and Mg+COL(10 mM Mg^2+^/Col I-coating group)

Figure 3 showed that there was no significant difference in cell morphology between NC (control group), Mg (10 mM Mg^2+^group), COL (Col I-coating group) and COL+Mg (10 mMMg^2+^/Col I-coating group). Under the microscope, there was no obvious phenomenon of apoptosis such as nuclear disintegration in deep nuclear staining.

### Cell adhesion

**Figure 4:**
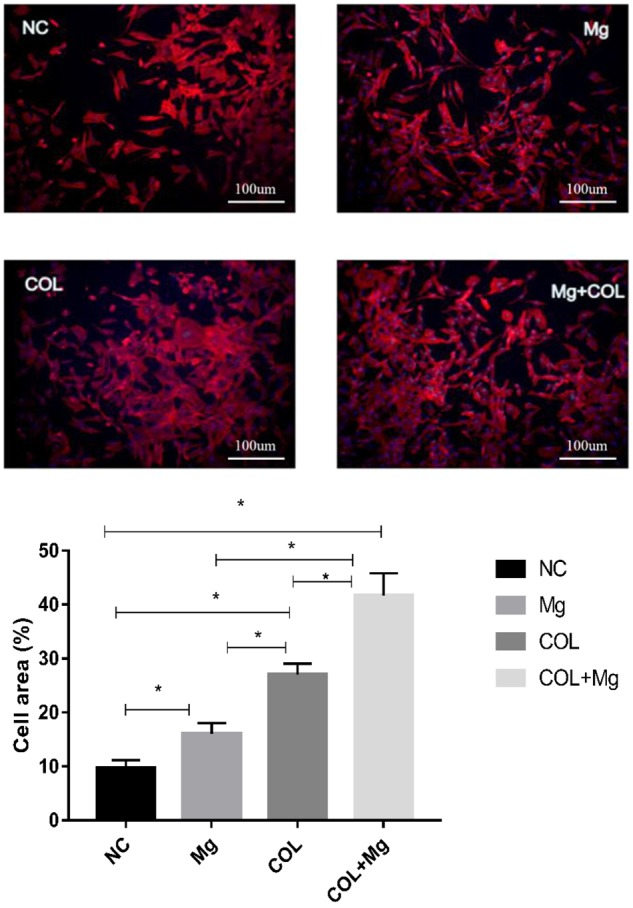
The cytoskeleton of osteoblasts were stained in NC (control group), Mg (10 mMMg^2+^ group), COL (Col I-coating group) and Mg+COL (10 mM Mg^2+^/Col I-coating group) (scale bar: 100 μm). Actin filaments were shown red and the nucleus were shown blue. One-way ANOVA (*n* = 3 per treatment group). *P < 0.05 versus control group

Figure 4 indicated that in the control group, osteoblasts grew flat and the extension was not obvious; however, in 10 mM Mg^2+^ group, Col I-coating group and 10 mM Mg^2+^/Col I-coating group, osteoblasts were densely distributed, full-bodied and stretch-extended, and clear, distinct actin filaments can be seen. In particular, 10 mM Mg^2+^/Col I-coating group of osteoblasts were densely distributed. Image J software was used to quantify acreage of the cell growth.

### Alkaline phosphatase activity

**Figure 5:**
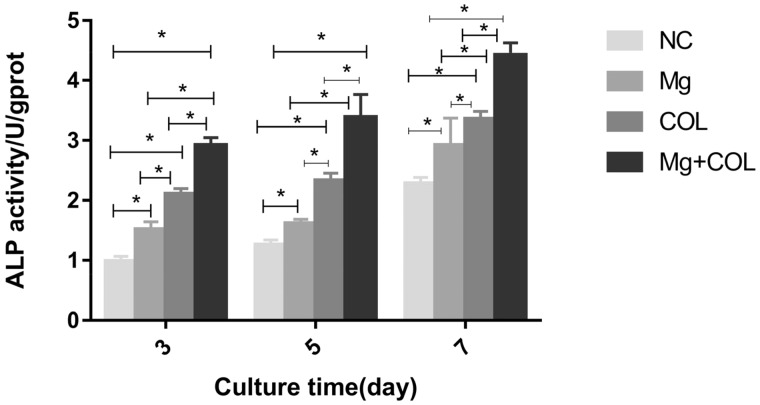
The ALP activity of NC (control group), Mg (10 mM Mg^2+^group), COL (Col I-coating group), Mg+COL (10 mM Mg^2+^/Col I-coating group) after incubation of MC3T3-E1 cells for 3, 5 and 7 days. One-way ANOVA (*n* = 5 per treatment group). *P < 0.05 versus control group

Figure 5 indicated that the viability of MC3T3-E1 cells cultured in different groups after 3, 5 7 days of incubation, the results indicated that compared with control group, 10 mM Mg^2+^ group and Col I-coating group, 10 mM Mg^2+^/Col I-coating group apparently promoted the viability of MC3T3-E1 cells. There were significant differences between all other groups (*P* < 0.05）. Especially 10 mM Mg^2+^/Col I-coating group significantly great promoted cell viability.

### Mineralization of ECM

**Figure 6:**
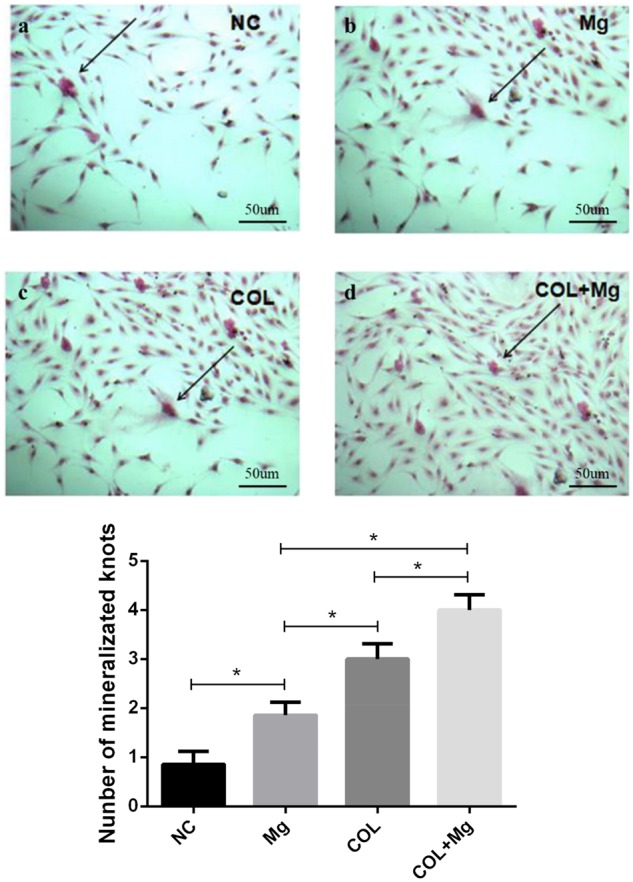
After incubation of 18 days, (**a**) the NC (control group), (**b**) Mg (10 mM Mg^2+^ group), (**c**) COL (Col I-coating group), (**d**) COL+Mg (10 mM Mg^2+^/Col I-coating group) of mineralization of ECM (scale bar: 50 μm)

To analyse the effects of different groups using Extracellular matrix mineralization experiment after 18 days of incubation. We used Alizarin red staining, Fig. 6 showed that (a) control group, (b) 10 mM Mg^2+^ group, (c) Col I-coating group, (d) 10mMMg^2+^/Col I-coating group. Compared with control group, other three groups apparently increased the mineralization knot of ECM. There were significant differences between all groups, especially 10 mM Mg^2+^/Col I-coating group significantly promoted the mineralization of ECM.

### Western blotting

**Figure 7:**
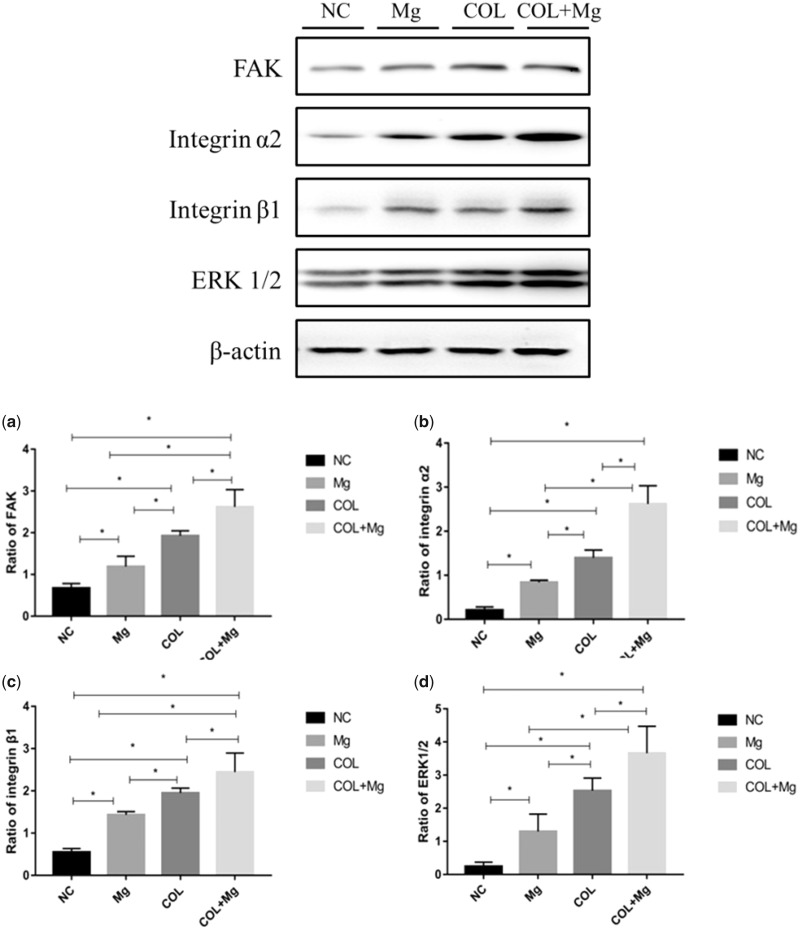
After 7 days of incubation, the NC (control group), Mg (10 mM Mg^2+^ group), COL (Col I-coating group), COL+Mg(10 mM Mg^2+^/Col I-coating group) that protein expression levels of FAK (**a**), integrin α2 (**b**), integrin β1 (**c**), ERK1/2 (**d**). One-way ANOVA (*n* = 3 per treatment group). *P < 0.05 versus control group

The different groups of protein levels (FAK, α2, β1 and ERK1/2) were tested by western blotting. Compared with the NC (control group), Mg(10 mM Mg^2+^ group), COL (Col I-coating group) and COL+Mg(10 mM Mg^2+^/Col I-coating group), Western blotting data indicated that protein expression levels of FAK, integrin α2, integrin β1 and ERK1/2 (a–d) increased obviously. There were significant differences between all groups, especially 10 mM Mg^2+^/Col I-coating group that had the highest protein levels of expression (Fig. 7).

### Expression of osteogenic genes

**Figure 8:**
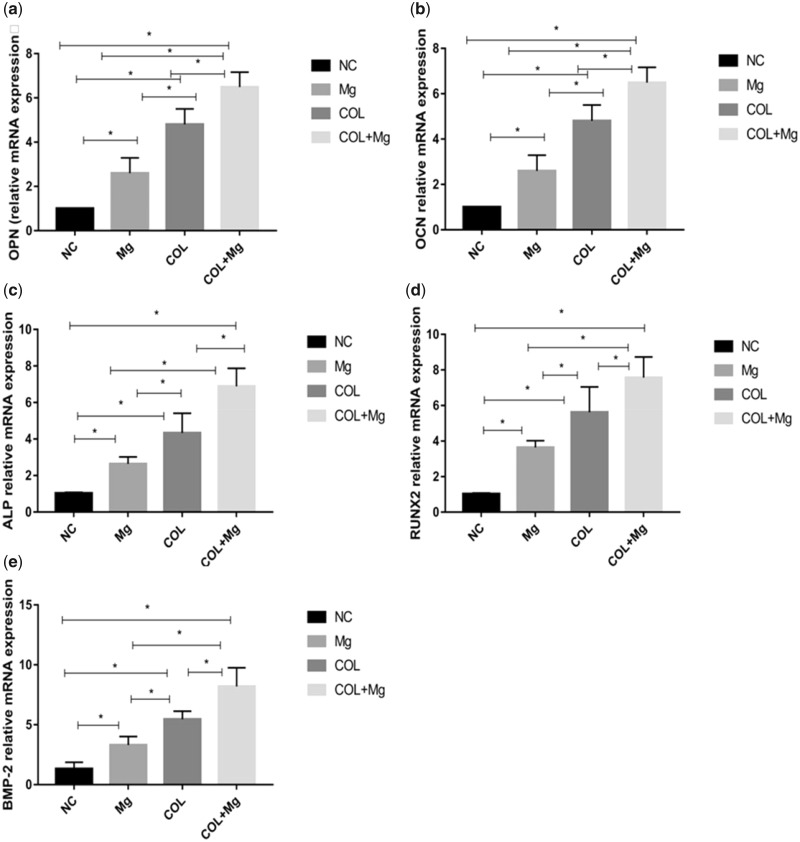
The NC (control group), Mg (10 mM Mg^2+^ group), COL (Col I-coating group), COL+Mg (10 mM Mg^2+^/Col I-coating group) that osteogenic-related genes expression levels of OPN (**a**), OCN (**b**), ALP (**c**), RUNX2 (**d**), BMP-2 (**e**) at 7 days were quantified by real-time PCR. One-way ANOVA (*n* = 3 per treatment group). *P < 0.05 versus control group

We quantified the osteogenic-related genes expression levels of OPN, OCN, ALP, RUNX2 and BMP-2 at 7 days by real-time PCR and the results were presented in Fig. 8. It was indicated that 10mMMg^2+^ and Col I, respectively, stimulated the osteogenic-related gene expression. Additionally, compared with control group, the osteogenic-related genes expression levels of OPN (a), OCN (b), ALP (c), RUNX2 (d) and BMP-2 (e) had arrived at the highest genes expression in 10 mM Mg^2+^/Col I-coating group. There were significant differences between all groups. One-way ANOVA (*n* = 3 per treatment group). *P < 0.05 vs. control group.

## Discussion

It is well known that most cellular processes rely on the formation of interactions between cells and extracellular matrices (ECMs). The key contributing factor to these interactions is integrins [19]. Integrins are integral membrane proteins that mediate cell matrix and cell-cell adhesion. Integrins mediate cell adhesion to collagen through magnesium-dependent interactions and it can be used as messenger conversion signals to initiate downstream cascades [20]. Many studies have showed that Mg^2+^ played a dual role in the integrins-collagen interaction and Mg^2+^ promoted osteoblasts adhesion through integrins and activated focal adhesion kinase (FAK) [21, 22]. FAK is a key component of the integrins-mediated signaling pathway. FAK act as a signaling molecule that transduces integrins receptor signaling through the intracellular protein cascade to participate in the adhesion process. FAK is considered to be the basic molecule of integrins dependent signal transduction pathway [23, 24]. In fact, the integrinα2β1-FAK-ERK (MAPK) signaling pathway was widely present in stromal cells [25, 26]. Moreover, MAPK/ERK is an important signaling pathway regulating bone development, bone remodeling and bone metabolism through promoting the expression of osteogenic-related genes [27, 28]. Some studies also showed Mg^2+^ promoted adhesion of osteoblasts, proliferation and differentiation by activating PI3K/Akt signaling pathway and Mg^2+^ participated in PI3K/Akt signaling pathway through ion channel functional protein kinase TRPM7 [13, 29]. There may be cross-activation between the PI3K/Akt signaling pathway and the ERK pathway. The osteogenesis mechanisms of Mg^2+^ need to explore in the future.

In our previous studies, we added additional MgCl_2_ solution to the medium, which is a neutral salt, however MgCl_2_ solution cannot change the pH of the medium. We also tested the activity of Mg^2+^ in the medium and medium showed the appropriate concentration. We compared the 6 mM −18mMMg^2+^, 10mMMg^2+^ promoted cells adhesion proliferation and differentiation, which was consistent with previous studies [13]. Therefore, in our present study, we investigated how 10 mM Mg^2+^ mediate integrin α2 and integrin β1-Col I binding and 10 mM Mg^2+^ bind Col I via integrin α2β1-FAK-ERK1/2 protein-coupled receptor pathway. To clarify the biological behavior effect of 10 mM Mg^2+^/Col I coating, we performed MTT, ALP, DAPI, Alizarin red staining and Rhodamine B-isothiocyanate (RITC)-labeled phalloidin experiments. We detected the expression of osteogenic-related genes (Runx2, ALP, OCN, OPN and BMP-2) by RT-PCR and the expression of signaling pathway proteins (integrin α2, integrin β1, FAK and ERK1/2) by Western blotting. By MTT, ALP and Alizarin red staining detections, we found that 10 mM Mg^2+^ group, Col I-coating group, 10 mMMg^2+^/Col I-coating group, respectively, promoted the proliferation and differentiation of osteoblasts, especially 10 mM Mg^2+^/Col I-coating group.

Moreover, our studies also showed cells adhesion proliferation and differentiation were higher in Col I-coating group and 10 mM Mg^2+^/Col I-coating group, especially 10 mM Mg^2+^/Col I-coating group had significantly difference. So, we speculated that Col I-coating materials had a good effect on the attachment, growth activity and function of osteoblasts and it play an important role in cell biological activity, cell compatibility and osteoinductivity and it is widely used in the field of biomedicine [30, 31]. This results also showed that Mg^2+^ in integrin-collagen binding had an important role and promoted integrin α2β1-Col I binding. This may be due to the fact that Col I-binding integrin are present in the a-subunit, inserted into the A domain, called the I domain. Mg^2+^ bind to metal ion-dependent adhesion sites on the I domain to mediate integrin α2β1-Col I binding. Our findings were in agreement with previous studies [32–34].

**Figure 9:**
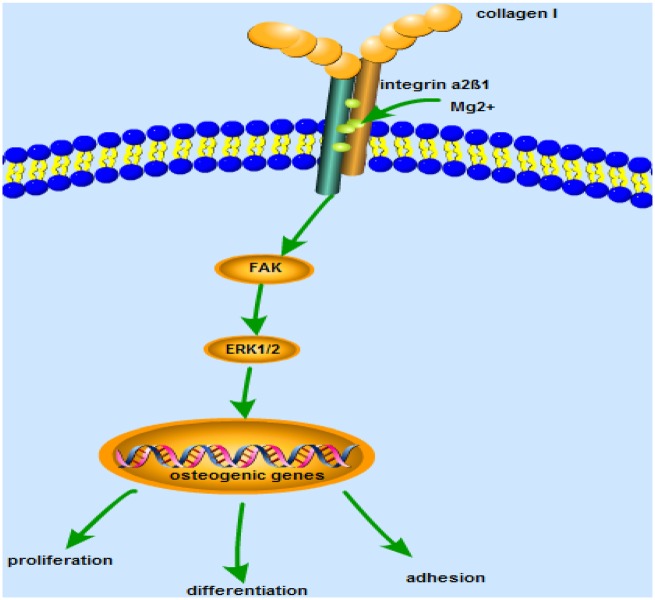
The potential mechanisms of integrinα2β1-FAK-ERK1/2 signaling in MC3T3-E1 cells

The major strength of this study was to prove the osteogenesis mechanisms of Mg^2+^-mediated integrin α2β1-Col I binding. However, our studies have some limitations. First, we have formalized the possible mechanisms of integrin α2β1-FAK-ERK1/2 signaling *in vitro* (Fig. 9) [35–37]. But the mechanisms of biological activity provided by this method have not been fully elucidated. Second, we used MC3T3-E1 cells for *in vitro* studies. But the *in vitro* simulated environment was different from *in vivo* studies, the degradation of Mg^2+^ concentration affected the environment inside the receptor. Its degradation rate was uncontrollable. At present, preliminary research on the mechanism, safety and effectiveness of the material was necessary. Third, it was difficult to assess the effects of surface topography. These provide new ideas for the future of magnesium-based composites and provide development prospects for better application in clinical practice. This still needs to be discussed in the future.

## Conclusions

In summary, our study demonstrated that the 10 mM Mg^2+^/Col I-coating play a critical role in up-regulating the MC3T3-E1 cells activity. Moreover, Mg^2+^ played a dual role in integrin α2β1-Col I to promote the biological behavior of MC3T3-E1 cells. The potential mechanisms of this specific performance may be through activating via integrin α2β1-FAK-ERK1/2 protein-coupled receptor pathway. However, the underlying osteogenesis mechanisms of Mg^2+^/Col I not fully understood. We need a further research on the degradation and osteogenesis mechanisms of magnesium-based composites.
